# Cellular Contractility Requires Ubiquitin Mediated Proteolysis

**DOI:** 10.1371/journal.pone.0006155

**Published:** 2009-07-14

**Authors:** Yuval Cinnamon, Oren Feine, Helfrid Hochegger, Alexander Bershadsky, Michael Brandeis

**Affiliations:** 1 The Department of Genetics, The Silberman Institute of Life Sciences, The Hebrew University of Jerusalem, Givat Ram, Jerusalem, Israel; 2 Sussex Centre for Genome Damage and Stability, University of Sussex, Brighton, United Kingdom; 3 Department of Molecular Cell Biology, The Weizmann Institute of Science, Rehovot, Israel; University of Birmingham, United Kingdom

## Abstract

**Background:**

Cellular contractility, essential for cell movement and proliferation, is regulated by microtubules, RhoA and actomyosin. The RhoA dependent kinase ROCK ensures the phosphorylation of the regulatory Myosin II Light Chain (MLC) Ser19, thereby activating actomyosin contractions. Microtubules are upstream inhibitors of contractility and their depolymerization or depletion cause cells to contract by activating RhoA. How microtubule dynamics regulates RhoA remains, a major missing link in understanding contractility.

**Principal Findings:**

We observed that contractility is inhibited by microtubules not only, as previously reported, in adherent cells, but also in non-adhering interphase and mitotic cells. Strikingly we observed that contractility requires ubiquitin mediated proteolysis by a Cullin-RING ubiquitin ligase. Inhibition of proteolysis, ubiquitination and neddylation all led to complete cessation of contractility and considerably reduced MLC Ser19 phosphorylation.

**Conclusions:**

Our results imply that cells express a contractility inhibitor that is degraded by ubiquitin mediated proteolysis, either constitutively or in response to microtubule depolymerization. This degradation seems to depend on a Cullin-RING ubiquitin ligase and is required for cellular contractions.

## Introduction

Cellular contractility and the ability of cells to change their shape are prerequisites for many biological phenomena such as cytokinesis, movement, differentiation and substrate adherence. These changes in cell shape are achieved by modulation of the cytoskeleton, most importantly the actin cytoskeleton, through forces generated by the actomyosin network. This network is regulated mainly through the activity of proteins from the Rho-GTPase family that regulate both actin nucleation and myosin activity through downstream effectors such as mDia and ROCK (Rho kinase), respectively (reviewed by [1], [2], [3]). Cell contractility is typically achieved by localized activation of Myosin II Light Chain (MLC) by its phosphorylation on Ser19. This phosphorylation, which causes contractions of the actin network by Myosin II is regulated by various MLC kinases and Myosin phosphatase (MYP). This site is phosphorylated by MLCK and ROCK [4], ZIP kinase [5] and citron kinase [6]. In addition to direct phosphorylation of Ser19, ROCK also phosphorylates and inactivates myosin phosphatase (MYP) enabling the maintenance of Ser19 phosphorylation. Inhibition of ROCK kinase inhibits contractility and Ser19 phosphorylation [7].

Less is known about the diverse upstream pathways through which RhoA acquires information from the cell's external and internal environments. It has been known for many years that depolymerization of microtubules leads to elevation of cell contractility, suggesting an inhibitory effect of microtubules on the actin cytoskeleton [8], [9]; reviewed by [10], [11]). It has also been shown that the microtubule polymerization inhibitor nocodazole induces RhoA activation [12]. This inhibition leads to the activation of MLC through ROCK by the release of the MAP GEF-H1 [13], [14].

RhoA is crucial for cytokinesis, where its local activation at the cell cortex determines the positioning of the cleavage furrow [15], [16], [17]. The major activator of RhoA in cytokinesis is the proto-oncogene RhoGEF Ect2 [18]. Inhibition of Ect2 leads to failure of cytokinesis and to binucleated cells ([19] and our unpublished results). Microtubules have been proven to have a crucial role in regulating cleavage furrow positioning, but the mechanism and microtubule effect on this process are still a matter of debate [20].

This project was initiated by our observation that cells arrested with nocodazole in prometaphase undergo vigorous contractions. We used time lapse microscopy to study these contractions and observed that they are not cell cycle specific and take place in non adhering cells throughout the cell cycle. These contractions were indeed sensitive to a variety of inhibitors of the RhoA pathway that we applied such as treatments with the exoenzyme C3 transferase, knockdown of Ect2 activity, expression of dominant negative RhoA and inhibition of ROCK with Y27632. We further showed that the RhoA-GEF Ect2, known to play a role in cytokinesis, also regulates contractions, at least as far as early mitotic cells are concerned.

The question how microtubule depletion activates the RhoA pathway remains a major unanswered issue. Given the major role of ubiquitination in most cellular events, we tested whether contraction activation requires ubiquitination. We used a proteasomal inhibitor and a cell line with temperature sensitive ubiquitin activating enzyme (E1). To our surprise we observed that ubiquitination and proteasomal degradation are required not for the activation but for the inhibition of contractility. The largest group of ubiquitin ligases in the cell constitutes of the Cullin-RING type. This vast group comprises among others the seventy different SCF (Skp1, Cul1, F-box proteins) complexes encoded by the human genome, and possibly more then a hundred Cul3-BTB based ligases [21]. To test whether such an ubiquitin ligase is involved in inhibition of contractility we took advantage of a cell line with a temperature sensitive neddylation pathway [22], [23]. Nedd8 is an ubiquitin like protein that gets covalently conjugated to lysine residues. Unlike ubiquitin it does not seem to form chains or lead to proteolysis. So far the only known substrates of neddylation are Cullins, subunits of the Cullin-RING ubiquitin ligases. Cullin neddylation prevents the binding of the CAND1 inhibitor and is essential for the activity of these ligases [24]. We show that myosin II light chain Ser19 phosphorylation, the most direct downstream event required for all types of contractility, strongly depends on proteolysis, ubiquitination and neddylation.

## Results

### Depolymerization of microtubules induces contractility in non-adhering cells

The microtubule cytoskeleton inhibits contractility of adherent cells in interphase [8], [9], probably by sequestering the microtubule associated protein GEF-H1 [13], [14]. Enhanced contractility is manifested by increased number and size of focal adhesions [25]. We studied the role of microtubules in the inhibition of contractility of non-adhering cells - cells that grow constitutively in suspension and adherent cells that were detached artificially or became detached during mitosis.

Nocodazole impairs the capacity of α-tubulin to polymerize and rapidly shifts the dynamic equilibrium of microtubules to a de-polymerized state (Movie S1). We chose chicken DT40 B-cells that grow in suspension (Movie S2A), to test the effect of microtubule depolymerization on contractility. Movie S2B shows that treatment of these cells with nocodazole rapidly led to contractions of the entire cell population. This suggested that contractions are not cell cycle dependent. Indeed DT40 cells synchronized at various stages of the cell cycle all underwent contractions upon nocodazole treatment (data not shown).

**Figure 1:**
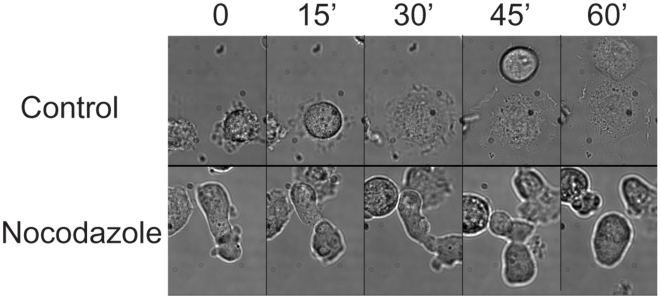
Detached adherent cells contract in a microtubules dependent manner. Unsynchronized NIH3T3 cells were trypsinized and plated in glass bottom dishes with (bottom panels) or without nocodazole (top panels). While control cells flattened within 30–60 minutes, nocodazole-treated cells failed to re-attach for more than 2 hours and during this time they continued to contract vigorously.

We wondered how adherent cells that become detached behave upon microtubule depolymerization. We therefore detached NIH3T3 cells from tissue culture dishes and observed their attachment to glass cover slips in the presence or absence of nocodazole. Without nocodazole the cells attached to the glass within one hour while in the presence of nocodazole they failed to attach for more than two hours and underwent vigorous contractions (Figure 1 and Movie S3).

**Figure 2:**
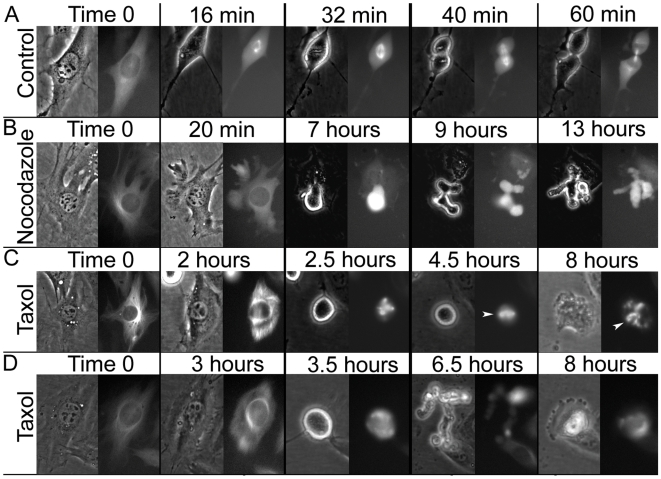
Mitotic NIH3T3 cells contract in a microtubule dependent manner. NIH3T3 cells stably expressing mCherry-α-tubulin were treated with various drugs that affect microtubule dynamics. Left panels phase, right panel mCherry-α-tubulin. A. An untreated cell undergoes mitosis within an hour (selected time points taken from Movie S4A). B. The effect of nocodazole on the microtubule network in interphase and mitosis. Time 0 shows the microtubule network, which has been disrupted following nocodazole addition (20 minutes, see also Movie S1). At 7 hours the cell entered mitosis and immediately contracted for the following 6 hours (selected time points taken from Movie S4B). C. Shows a cell with a spindle formed in the presence of taxol (80% of the treated cells). Taxol was added an hour after time 0. A thick and rigid microtubule pattern was observed within one hour. Upon mitotic entry (2.5 hours) the cell formed a spindle that was apparent for the next 5.5 hours (arrowhead). During this time the cell did not contract. Only after 8 hours the cell flattened while the spindle was still visible (arrowhead). D. Represents a less common effect of taxol (20% of the treated cells), which does not lead to spindle assembly. Taxol was added an hour after time 0 and the cell entered mitosis at 3.5 hours. This cell did not form a spindle and furrowed for 4.5 hours until it flattened at 8 hours.

During mitosis the microtubule cytoskeleton undergoes a radical change of its shape, composition and dynamics. We thus wondered if it retains its capacity to inhibit cellular contractions. Such an observation would be of significance for the capacity of cells to undergo furrowing in cytokinesis. To follow the behavior of the spindle we generated a NIH3T3 mouse fibroblast cell line that stably expresses α-tubulin fused to the mCherry [26] fluorescent protein (Figure 2 and Movie S1). We treated these cells with nocodazole and followed them by time lapse microscopy. Figure 2B and Movie S4B show that as long as cells were in interphase they remained attached to the matrix and did not visibly contract. Upon reaching prometaphase they detached from the matrix, as expected from mitotic cells, and initiated contractions at multiple random locations. Cells arrested at this stage by the spindle assembly checkpoint (SAC) for several hours. We thus concluded that microtubule depolymerization induces contractility throughout the cell cycle.

We next asked whether microtubule stabilization would induce an inhibitory effect on contractility. This was achieved by inhibiting α-tubulin depolymerization with Paclitaxel (taxol). Treated cells exhibited thick and rigid microtubule bundles. Like in the case of nocodazole, taxol-treated cells arrested in prometaphase by the SAC. This arrest had either of two phenotypes. The majority of the cells exhibited rigid spindles, did not contract but arrested as motionless round mitotic cells for several hours until they flattened, presumably due to mitotic slippage [27] (Figure 2C). About a fifth of the cells, however, failed to assemble spindles and underwent contractions similar to those demonstrated by nocodazole treated cells (Figure 2D). The reason for that is unknown but we speculate that this is because taxol interferes with the dynamic instability of microtubules which in some cases resulted in the collapse of the microtubule cytoskeleton (Figure 2D timepoint 3.5 hours).

**Figure 3:**
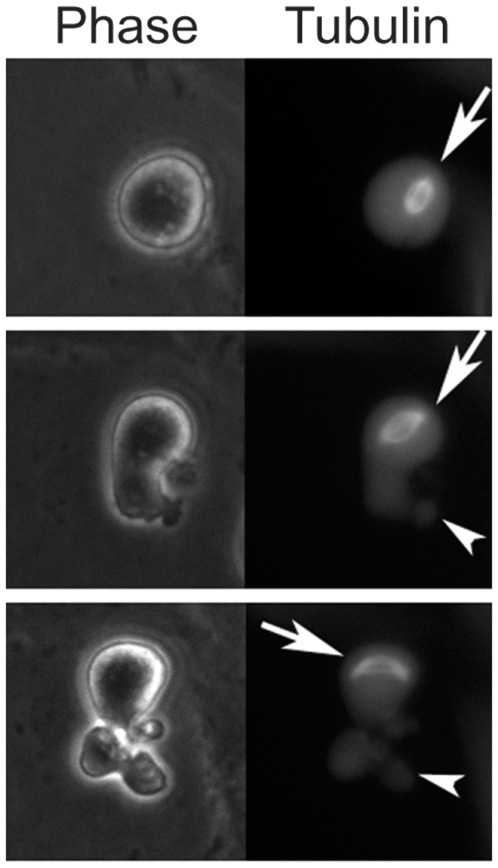
The mitotic spindle in cells arrested by nondegradable Cyclin B1 inhibits contractions in a spatial dependent manner. NIH3T3 cells stably expressing mCherry-α-tubulin were transfected with Cyclin B1-DM-GFP. This Figure shows frames of three selected time points taken from Movie S5A. Following mitotic entry and spindle formation (2 hours, arrow) a part of the cell, which was distal to the spindles (3.5 hours, arrowhead) started to contract, while a part of the cell that remained proximal to the spindle did not (5 hours).

The cells discussed so far were treated for several hours in interphase with the indicated drugs until they reached prometaphase. Since these drugs arrest the cells at the SAC, we sought an alternative method to arrest cells later in mitosis, allowing the spindles to form normally and test the instant effect of their de-polymerization. To this end we transiently transfected cells with an expression vector for a destruction box mutant of full length Cyclin B1-GFP (Cyclin B1-DM-GFP), which is not ubiquitinated by the APC/C and is thus not degraded. These cells arrested in mitosis either before metaphase or in anaphase [28], [29], [30]. Figure 3 and Movie S5A show that Cyclin B1-DM-GFP expressing cells formed a normal spindle and maintained it for a very long time (Figure 3 top panel). These cells did not slip through the SAC for at least 48 hours, demonstrating that SAC slippage occurs indeed due to APC/C specific Cyclin B1 degradation. When Cyclin B1-DM-GFP arrested cells were treated with nocodazole they promptly started to contract, and kept contracting for many hours (Movie S5B). Strikingly when the spindle in Cyclin B1-DM-GFP arrested cells drifted to one part of the cell, the part of the cell distal to the spindle promptly started to contract. When the spindle drifted back contractions stopped upon approach of the spindle (Figure 3 central and bottom panels and Movie S5A). We observed this kind of drifting and contractions of the distal part of the cell in 13 out of the 64 cells. Contractions were never observed in proximity to the spindle.

We further used monastrol, which arrest the cells in mitosis with mono-astral spindles without interfering with microtubule dynamics [31]. Monastrol treated cells did not contract but promptly started to do so upon treatment with nocodazole (table 1). The contractility we observed is thus not a mere effect of drugs interfering with microtubule dynamics, but occurs in regions of the cell that have become depleted of microtubules by other causes as well.

**Table 1:** Cell contractility is induced by microtubule depolymerization.

Cells	Treatment	Effect	Contractions	Figure	Movie	Quantization*
DT40	Nocodazole	Microtubule depolymerization	Yes		S2B	89% n = 70
Detached NIH3T3				1	S3	>97% n≈100
Mitotic NIH3T3				2B	S4B	85% n = 186
NIH3T3	Taxol	Microtubule stabilization	With spindle – No	2C		80% n = 57
			Without spindle - Yes	2D		20% n = 57
NIH3T3	Cyclin B1-DM	Metaphase/Anaphase arrest	Close to spindle – No Far from spindle - Yes	3	S5A	100% n = 64 20% n = 64
	Cyclin B1 DM +Nocodazole	Metaphase/Anaphase arrest+ Microtubule depolymerization	Yes		S5B	92% n = 135
NIH3T3	Monastrol	Monopolar spindles due to Eg5 inhibition and lack of centrosome separation	No			89% n = 65
NIH3T3	Monastrol+Nocodazole	Monopolar spindles followed by microtubule depolymerization	Yes			84% n = 71

* The percentage refers to the phenotype declared in the contractions column.

The results we report here show that cells that are not attached to a matrix will undergo contractions upon microtubule depletion whether they grow constitutively in suspension, are artificially detached in interphase, or physiologically in mitosis. We thus conclude that both the interphase microtubule cytoskeleton and the mitotic spindle inhibit cellular contractility. A detailed breakdown of these results and quantitative data is presented in table 1.

### Contractility induced by microtubule depolymerization depends on the RhoA pathway

**Figure 4:**
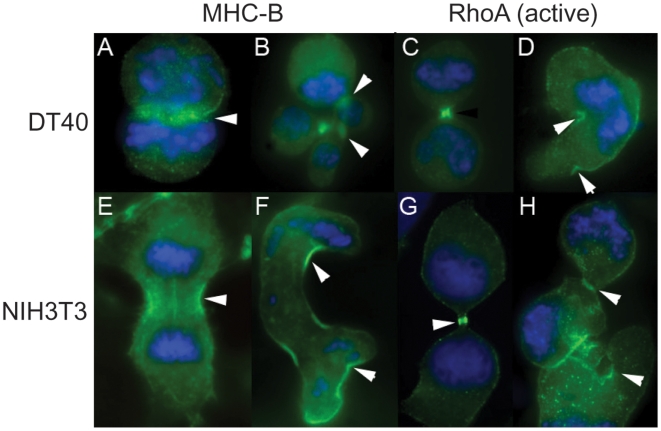
Myosin and active RhoA localize to invaginations formed by contractions. Myosin heavy chain – B (MHC-B) and active RhoA are localized to the cleavage furrow and midbody as well as to the contraction invaginations of nocodazole-treated mitotic cells (arrowheads). A–D, DT40 cells; E–H NIH 3T3 cells; A, E, C, G control untreated cells; B, D, F, H, nocodazole-treated cells.

**Figure 5:**
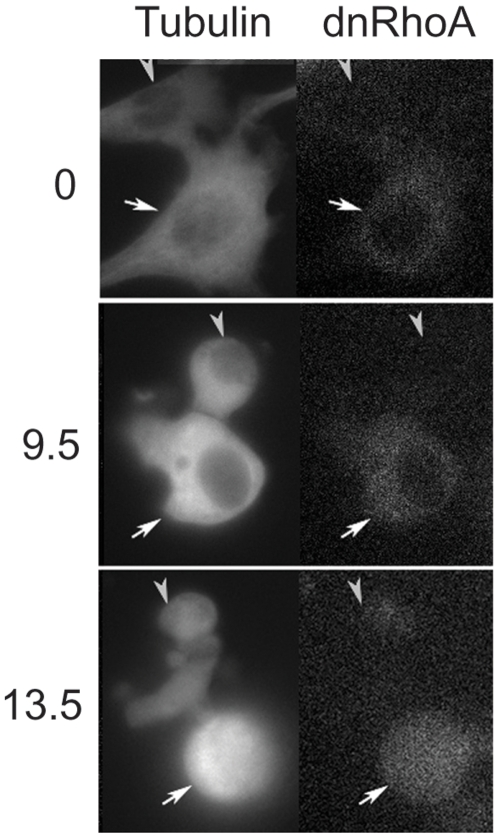
Inhibition of RhoA inhibits nocodazole-induced contractions. NIH3T3 cells stably expressing mCherry-α-tubulin were transfected with pEGFP-hRhoA T19N (*dn*RhoA-GFP) and treated with nocodazole. The left panel shows the mCherry−α-tubulin pattern and the right panel shows *dn*RhoA-GFP expression. Two cells can be seen: a *dn*RhoA-GFP transfected cell (arrow) and an untransfected cell (arrowhead). They both entered mitosis at about the same time (9.5 hours). While the *dn*RhoA-GFP expressing cell remained arrested for 5 hours and did not contract, the non transfected adjacent cell contracted vigorously (see also Movie S6).

Cellular contractility by actomyosin depends on the RhoA signaling pathway. We wanted to verify that the contractions induced by microtuble depolymerization also depend on this pathway. Cells were immuno-stained for RhoA and Myosin. Figure 4 shows that indeed Myosin and RhoA localized to the furrows that formed in contracting cells in a manner similar to their localization to the cytokinetic cleavage furrow. The RhoA localized to these furrows is likely to be in its active form [32], [33]. We used a variety of methods to inhibit the different stages of the RhoA pathway. RhoA was inhibited by the exoenzyme C3 transferase and ROCK was inhibited by the Y27632 inhibitor. In both cases contractions in response to nodocazole treatment were completely eliminated (table 2). We further transfected cells with a vector expressing the dominant negative RhoA T19N mutant. In the experiment shown in Figure 5 and Movie S6 an expressing and a non-expressing cell are shown side by side. The transfected cell did not contract while its non-expressing neighbor contracted vigorously. Finally we tested the effect of inhibition of the Ect2 GEF, which is known to activate RhoA in cytokinesis. Both a dominant negative expression vector and relatively modest siRNA mediated knockdown of Ect2 (60%, Figure S1A-C) completely eliminated contractions. Figure S1D shows that this relatively modest knockdown was also sufficient for perturbing cytokinesis resulting in many binucleated cells. All these experiments, summarized in table 2, show that nocodazole induced contractions fully depend on the RhoA signaling pathway. They also indicate that these contractions are not “membrane blebbing” events but cortical contractions involving RhoA and actomyosin activation.

**Table 2:** Cell contractility requires the RhoA pathway.

Cells	Nocodazole+	Microtubule depolymerization+	Contractions	Quantization*
NIH3T3			Yes	85% n = 186
NIH3T3	Y27632	Inhibition of ROCK	No	91% n = 54
NIH3T3	Exoenzyme C3 transferase	Inhibition of RhoA	No	70% n = 95
NIH3T3**	*dn*RhoA	Inhibition of RhoA	No	93% n = 40
NIH3T3	ECT2 knockdown	Inhibition of Ect2	No	87% n = 80
NIH3T3	*dn*ECT2 siRNA knockdown	Inhibition of Ect2	No	
DT40			Yes	89% n = 70
DT40	Y27632	ROCK inhibition	No	85% n = 46
DT40	Exoenzyme C3 transferase	RhoA inhibition	No	74% n = 50

* The percentage refers to the phenotype declared in the contractions column.

** Figure 5 and Movie S6.

### Contractility requires ubiquitin dependent proteolysis by a Cullin-RING ubiquitin ligase

**Figure 6:**
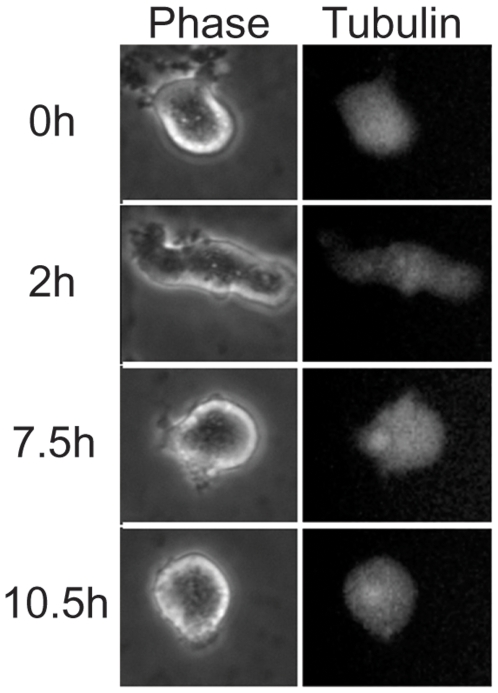
Cellular contractility requires proteasome-mediated degradation. This NIH3T3 cell was treated with nocodazole, entered mitosis and contracted prior to the addition of MG132 at time 0. The cell ceased to contracted after several hours (7.5 and 10.5 hours, see also Movie S7A).

Ubiquitin mediated proteolysis plays a role in most cellular events. We observed that ubiquitin is localized to the furrows formed in the absence of microtubules as well as in the cleavage furrow during cytokinesis (our unpublished data) and we wondered whether ubiquitin mediated proteolysis is also involved in regulation of contractility. We therefore treated cells with nocodazole together with the proteasome inhibitor MG132. Figure 6 and Movie S7A show that MG132 treatment completely abolished contractions of prometaphase arrested cells. Strikingly, when cells that were already arrested with nocodazole in mitosis and undergoing contractions were treated with MG132 they stopped to contract within 7 hours. We wondered whether cells stopped contracting due to a non specific irreversible harm caused by MG132. We therefore washed cells that were arrested for 15 hours with MG132 and nocodazole and re-plated them into fresh medium. Movie S7B shows that these cells resumed contractility and attached to the matrix within two hours precluding an irreversible harm. These findings suggest the existence of one, or more, proteasomal substrates that inhibit cell contractility.

Proteasomal inhibition leads to cell cycle arrest at various phases. The requirement for proteasomal activity for contractions could thus be due to cell cycle effects. To address this possibility, we treated unsynchronized DT40 cells with nocodazole together with MG132. This double treatment completely abolished contractions regardless of cell cycle stage (table 3). The requirement for proteasomal degradation is thus not restricted to mitotic cells and does not require arrival at specific cell cycle phases.

**Table 3:** Cell contractility requires ubiquitin mediated degradation.

Cells	Treatment Nocodazole +	Effect Microtubule depolymerization+	Contractions	Figure	Movie	Quantization*
NIH3T3	MG132	Proteasome inhibition	No	6	S7	80% n = 35 88% n = 17
A31N-wt	39.5^0^		Yes		S8B	92% n = 48**
A31N-ts20	34^0^		Yes		S8C	90% n = 51**
A31N-ts20	39.5^0^	E1 inhibition	No		S8D	94% n = 48
E36-wt	39.5^0^		Yes		S9B	92% n = 50**
E36-ts41	34^0^		Yes		S9C	90% n = 48**
E36-ts41	39.5^0^	Neddylation inhibition	No		S9E	94% n = 46
E36-wt	39.5^0^ ***		Yes		S9F	>97% n≈100
E36-ts41	39.5^0^ ***	Neddylation inhibition	No		S9F	>97% n≈100
DT40	MG132	Proteasome inhibition	No			81% n = 67

* The percentage refers to the phenotype declared in the contractions column.

** Mitotic cells that vigorously furrowed for any length of time.

*** Detached cells were seeded on glass bottom culture plate.

To verify the need for ubiquitin-mediated proteolysis in a non-drug dependent manner we used the balb/c 3T3 derived mouse fibroblast cell lines A31N-wt and A31N-ts20. The latter expresses a thermo-sensitive E1 ubiquitin activating enzyme that becomes inactive at the restrictive temperature of 39.5°C [34]. Wild type A31N cells cultured either at 34°C (not shown) or 39.5°C (Movie S8A) divided normally. Upon addition of nocodazole to the growth medium the cells arrested at the SAC and started contracting (Movie S8B). A31N-ts20 cells behaved like wild type A31N cells at 34°C (Movie S8C). However when A31N-ts20 mutants were grown at 39.5°C for 8–14 hours and treated with nocodazole the cells that reached mitosis arrested as round cells, did not contract and did not flatten (Movie S8D). These findings indicate that the ubiquitination pathway is required for contractility induced by microtubule depolymerization.

Cullin-RING complexes are the largest group of ubiquitin ligases and are involved in the regulation of many cellular pathways [21]. To test whether they are required for contractility we used the hamster cell line E36-ts41 [23], [35], which has a temperature sensitive neddylation pathway [22]. Nedd8 is an ubiquitin-like protein that is covalently conjugated to members of the Cullin subunit of E3 ligases. This modification is essential for the activation of Cullin-RING E3s [24]. We used the E36-ts41 and its parental wild type E36-wt line to test if a Cullin-RING E3 mediates the ubiquitination that is required for contractions. Wild type E36 cells divided normally both at 34°C (not shown) and 39.5°C (Movie S9A). Upon treatment with nocodazole these cells arrested at the SAC, contracted and flattened within a few hours (Movie S9B). Mutant E36-ts41 behaved at 34°C like wild type cells (Movie S9C) and at 39.5° they arrested and did not divide (Movie S9D). When the incubation temperature for E36-ts41cells was shifted from 34°C to 39.5°C and nocodazole was added, the cells that entered mitosis from 8 hours and onwards did not contract (Movie S9E). Interestingly the minimal incubation time at the restrictive temperature, required for the elimination of contractions, was almost identical for both the ts20 and the ts41 cells (8 hours) and for MG132 (7 hours). The results of these experiments and quantitative data are summarized in table 3.

**Figure 7:**
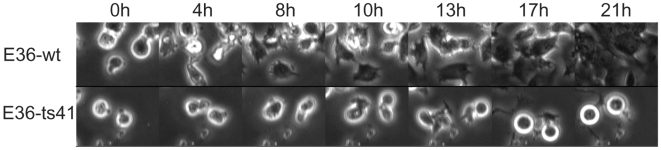
Contractility requires neddylation. Trypsinized E36-wt and E36-ts41 (upper and lower panels respectively) cells, normally incubated at 34°C, were treated with nocodazole and plated in glass bottom dishes at 39.5°C. Control E36-wt cells vigorously contracted for 8–10 hours and then, within the next 13 hours of incubation, and gradually flattened (10, 13, 17, 21 hours). E36-ts41 cells contracted as the control cells for the first 10 hours and then gradually contraction ceased. This 10 hours time point corresponds to the time necessary for the thermosensitive mutation to become active and reflects the residual neddylation which existed prior to the temperature shift. Moreover, these round and motionless cells failed to re-adhere the plate (time points 13, 17, 21 hours and Movie S9F).

These mutant cells enabled us also to test the requirement for neddylation for contractility in detached interphase cells. We plated E36-wt and E36-ts41 cells on glass in the presence of nocodazole at the restrictive temperature of 39.5°C. E36-wt cells contracted vigorously like the NIH3T3 cells shown in Figure 1 and Movie S3, and began to flatten after 4 to 8 hours (Figure 7 upper row and left panel of Movie S9F). E36-ts41 contracted initially for about 10 hours and then ceased to contract but arrested as round cells and did not flatten (Figure 7 lower row and right panel of Movie S9F), demonstrating that these contractions are neddylation dependent. This observation shows that neddylation is required for contractions in a cell cycle independent manner. In a control experiment without nocodazole both E36-wt and E36-ts41 cells plated on glass incubated at either temperature flattened within 2–4 hours (data not shown).

### Phosphorylation of Ser19 of MLC requires proteolysis, ubiquitination and neddylation

**Figure 8:**
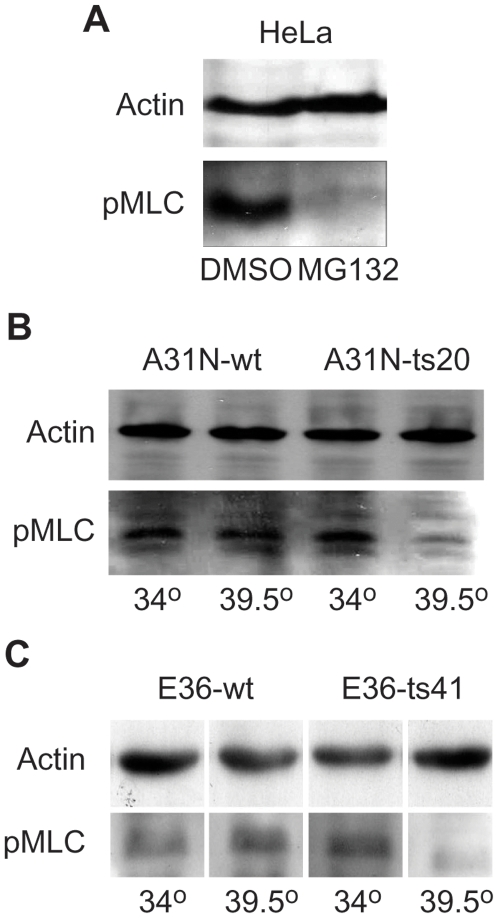
Proteolysis, ubiquitination and neddylation are required for MLC phosphorylation. A, HeLa cells were suspended by trypsinization and grown for five hours in suspension in plastic tubes in the presence of nocodazole with or without MG132. Cells were harvested and analyzed by western blot with anti-phosoho-myosin antibodies. B, A31N-wt and A31N –ts20 cells were grown over-night at the indicated temperatures. They were subsequently trypsinized and grown in suspension in plastic tubes for an additional 2 hours in the presence of nocodazole at the same temperatures. Cells were harvested and analyzed by western blot with anti-phosoho-myosin antibodies. C, E36-wt and E36-ts41 cells were grown over-night at the indicated temperatures. They were subsequently trypsinized and grown in suspension in plastic tubes for an additional 2 hours in the presence of nocodazole at the same temperatures. Cells were harvested and analyzed by western blot with anti-phosoho-myosin antibodies.

The most downstream regulatory event of cellular contractility is the activating phosphorylation of Ser19 of MLC. The data presented so far suggested that microtubules inhibit contractility and upon relief the contractility requires ubiquitin mediated proteolysis by a Nedd8 dependent pathway. In order to assess whether this requirement directly impinges on the phosphorylation of this site we used anti-phospho Ser19 MLC antibodies. We addressed this issue with all three treatments. First we analyzed the effect of inhibition of proteolysis in human HeLa cells. Cells were detached from the tissue culture dishes and treated with nocodazole for five hours with or without MG132. Preliminary experiments have shown that detachment induces Ser19 phosphorylation, which gets further enhanced by nocodazole. Figure 8A shows that Ser19 phosphorylation was almost completely eliminated by treatment of cells with MG132. Next we analyzed Ser19 phosphorylation in the mouse A31N-ts20 cell line at the permissive versus the restrictive temperature. Figure 8B shows that phosphorylation was drastically reduced in A31N-ts20 cells at the restrictive temperature. In the wild type control cells, Ser19 phosphorylation was invariant to temperature. Finally we performed a similar analysis in E36-ts41 cells and here too it is evident that Ser19 phosphorylation is much lower at the restrictive temperature of the mutant cells and not affected in wild type controls (Figure 8C). These results clearly show that proteolysis, ubiquitination and neddylation are required for Ser19 phosphorylation.

### Constitutively active RhoA overrides the requirement of ubiquitination for contractions

**Figure 9:**
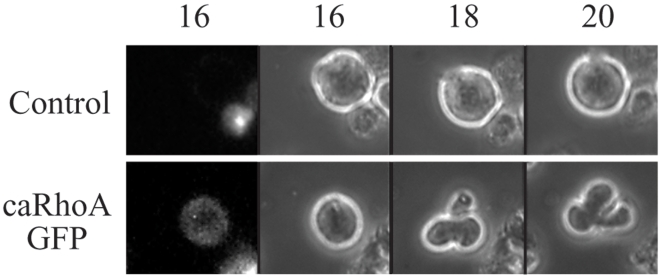
Neddylation is upstream of the RhoA pathway. E36-ts41 control cells or E36-ts41 mutant cells transfected with an expression vector to the constitutively active RhoA mutant Q63L-hRhoA fused to GFP (*ca*RhoA) (upper and lower rows respectably) were grown at 39.5°C in the presence of nocodazole. While control cells did not contract, *ca*RhoA-expressing cells contracted for 22 hours.

We have shown that contractions depend both on the RhoA pathway and on the activity of a Cullin-RING E3. To establish the relationship of these two pathways, we transfected E36-ts41cells with an expression vector for a constitutively active form of RhoA (Q63L-hRhoA) fused to GFP (*ca*RhoA-GFP) and cultured them at 39.5°C in the presence of nocodazole. Under these conditions protein degradation, which is mediated by Cullin-RING ubiquitin ligases, is blocked while the RhoA pathway is active. Figure 9 and Movie S10 show that untransfected cells did not contract, while *ca*RhoA-GFP expressing cells vigorously contracted. This suggests that RhoA activity is downstream to the Cullin-RING E3 and might be dependent on it for contracting. This experiment also verifies that loss of contractions in response to neddylation inhibition is a rather specific event and not a result of ATP depletion in cells.

We searched for potential candidate proteins that inhibit RhoA and contractility and that are degraded in response to microtubule de-polymerization. The RhoA inhibitor p190RhoGAP that is degraded during the cell cycle [33], could have been a suitable candidate. We observed however that p190RhoGAP is not degraded in response to microtubule depolymerization. These findings thus exclude it from being the inhibitor we are currently seeking (data not shown).

## Discussion

In this study we addressed the regulation of cellular contractility, a behavior of major importance for cell movement and proliferation. Contractility in response to microtubule depolymerization has been observed already 20 years ago [8], [9]. Our research has extended these observations to non-adhering cells and to cells during mitosis. These observations are in agreement with a recent report of contractions of non-adhering cells and of cell fragments [36].

We show that movement of the mitotic spindle can induce contractility also in the absence of drugs. This observation stresses the importance of the spindle in the inhibition of contractions, and is reminiscent of the effect observed in response to manual manipulations of the spindle of echinoderm embryos [15]. Such observations are of significance for the ongoing debate whether the cytokinetic furrow is actively induced by the spindle midzone, or by relaxation of inhibition of microtubules that take place at a place furthest away from the microtuble organizing centers [20], [37], [38]. While our results support the latter model, they do not directly contradict the first and the possibility that the mechanisms that regulates cytokinetic furrow initiation differ from the mechanism presented in this work.

Niiya et al [39] showed that Cdk1 inactivation in early mitosis can leads to precocious cytokinesis. Their observations however do not imply that Cdk1 inactivation is essential for contractions. We have shown here that contractions can take place anytime during the cell cycle, some of which will have low others high levels of Cdk1 activity. As a matter of fact most of the contractions described here took place in prometaphase arrested cells, which have high levels of Cdk1 activity.

We confirmed here that cellular contractility induced by microtubule depolymerization depends, as previously reported [40], [41], on the RhoA-ROCK pathway. The RhoA GTPase requires also a GEF for its activation. GEF-H1 has been implicated in contractility in the past [13], [14]. The RhoA-GEF Ect2 is considered to be specific for activation of RhoA in cytokinesis [18], [20]. We observed that Ect2 is essential for contractility during early mitosis. As Ect2 is present during all stages of the cell cycle [18] and our data (not shown), it could indeed control contractility also in interphase.

Our observation, that contractility requires ongoing ubiquitin mediated proteasomal degradation, is highly significant. Its requirement for neddylation suggests that degradation is most likely mediated by a Cullin-RING ubiquitin ligase. Our results imply that the cell is continuously synthesizing a protein that inhibits contractility and that must be degraded for cells to contract. Potapova et al. [42] have shown that, as long as Cdk1 activity is inhibited, proteasomal activity is not required for cytokinesis. In their experiments the proteasome was inhibited for less than an hour. All the approaches we used show that the re-accumulation and effect of the yet unknown contractility inhibitor take roughly seven hours. This response is relatively slow compared to other cellular events. As a matter of fact this time span is reminiscent of the time it takes tissue culture cells arrested prior to the restriction point [43] to exit G0 in response to stimulation by growth factors [44]. Such a prolonged inhibition of proteasomal degradation could potentially lead to non physiological stress and possibly to depletion of ATP. Experiments shown in Figure 9 and Movie S10 make such an explanation unlikely. Cells with an inhibited neddylation pathway expressing a dominant negative RhoA do not stop contracting like their untrasfected neighbors. This indicates that lack of ATP or a non specific effect are unlikely to lead to the cessation of contractions in these cells. Movie S7B shows that cells inhibited for a prolonged time in MG132 recover and resume contractile behavior once they are washed and transferred to MG132 free medium.

A particular intriguing possibility is that degradation can be modulated or inhibited by the cell under conditions where cells are not supposed to contract, to move, or to divide. It would be of interest to establish whether terminally differentiated cells in tissues that are neither supposed to divide nor to wander around still degrade this inhibitor.

**Figure 10:**
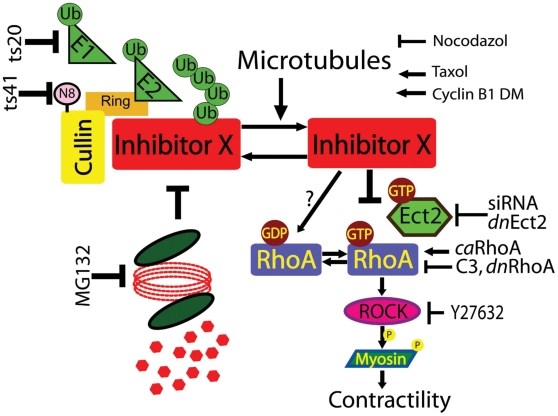
Hypothetical model of regulation of cell contractility. The model shows how microtubule stability is upstream of the inhibitor. Once microtubules are broken down or removed the inhibitor is degraded enabling the activation of the RhoA cascade. We included all the inhibitors and mutants we used to test this hypothesis.

The hypothetical model we propose described in Figure 10 suggests that this inhibitor is degraded in response to microtubule depolymerization. It is however also possible that degradation is ongoing and that the inhibitory mechanisms act in parallel. We show that this degradation directly impinges on the phosphorylation of Ser19 of Myosin II light chain. As ROCK and other kinases directly phosphorylate MLC Ser19, it is conceivable that the inhibitor acts upstream. Indeed expression of constitutively active RhoA managed to override this inhibition. We don't have however evidence that the inhibitor acts directly on the RhoA pathway.

The search for this inhibitor and the characterization of its degradation are a major challenge for future studies currently underway in our lab. Inhibition of such a mechanism by specifically developed drugs can be a useful and highly specific approach to inhibit cell movement and division, two hallmarks of metastasizing cancer cells.

## Materials and Methods

### Cells, cell culture and reagents

NIH-3T3, E36-ts41, E36, A31N and A31N-ts20 cell lines were maintained in Dulbecco's modified Eagle medium (DMEM, Gibco) containing 10% fetal calf serum, 10 u/ml Penicillin and 100 µg/ml Streptomycin (Biological Industries, Beit-Haemek). DT40 cells were maintained in RPMI-1640 (Gibco) containing 10% fetal calf serum, 3% chicken serum, 10 u/ml Penicillin, 100 µg/ml Streptomycin and 50 µM 2-mercaptoethanol (Merck). NIH3T3 and DT40 were grown at 37°C with 5% CO_2_. E36-wt, E36-ts41, A31N-wt and A31N-ts20 were grown either at 34°C or at 39.5°C (as described in Results) in 5% CO_2_.

The reagents were added to the growing media to reach the indicated final concentration as follows: nocodazole (Sigma) 2 µM (0.2 µM for HeLa cells), Taxol (Sigma) 5 µM, MG132 (Sigma) 0.1 µM (0.05 µM for HeLa cells), Cell permeable exoenzyme C3 transferase (Cytoskeleton) 7.5 µg/ml, Y27632 (Sigma) 15 µM. Ect2 siRNA (Santa Cruz Biotechnology sc-35259), non-specific-siRNA and siRNA delivery control (Mirus) were transfected using Lipofectamine 2000 (Invitrogene) according to manufacturer guidelines.

### DNA cloning and plasmid constructs

The pEF-Cyclin B1DM-GFP vector expressing non degradable full-length mouse Cyclin B1-GFP was previously described [45]. pEGFP-hRhoA T19N (*dn*RhoA) and pEGFP-hRhoA Q63L (*ca*RhoA) were a gift from S. Ravid. pEGFP-Ect2-N1 (*dn*Ect2) [46] was a gift of S. Narumiya.

pEF-mCherry-α-tubulin was made by Y. Oren by cloning *α-tubulin* downstream and in frame with the gene for the *mCherry* fluorescent protein [26] in the pEF-plink2 expression vector. All vectors were sequenced and verified. Transient transfections and stable lines were obtained by the CaPO_4_ co-precipitation method [47].

### Antibodies and fluorescent labeling

Rabbit anti-pMLC (Cell Signaling) and Goat anti-actin (Santa Cruz Biotechnology sc-1616) were used for western blotting. Mouse anti-RhoA (26C4) (Santa Cruz Biotechnology sc-418) diluted 1∶200, Rabbit anti-mMHC-B (S. Ravid) diluted 1∶200, Donkey anti-Mouse IgG (H+L) Cy2 conjugated diluted 1∶500, Donkey anti-Rabbit IgG (H+L) Cy2 (Jackson Immunoresearch). RhoA immunofluorescent labeling was done as recently described. For mMHC-B labeling, cells were fixed in 4% formaldehyde in PBS for 20 minutes and blocked with PBST. The slides were visualized either on an inverted IX70 Olympus microscope and captured with a CoolSnap HQ (Photometrics) digital camera, or on an Olympus FV1000 confocal microscope. The images were analyzed and assembled with Image pro plus 5.0 (Media Cybernetic), ImageJ (http://rsb.info.nih.gov/ij/) and Photoshop 7 (Adobe) software. Movies were assembled by Premier-Pro (Adobe).

### Time lapse microscopy

Cells were seeded on 35 mm tissue culture plates (Nunc) a day prior of filming. The cells were observed by an Axiovert 200 M microscope (Zeiss) equipped with a CO_2_ and temperature-controlled incubator (EMBL GP168) and SM1 motorized stage (Luigs & Neumann). Images were captured with LD32X/0.4 air or 40X/1.3 oil objective lenses using a SensiCam QE (PCO) digital camera and CompiC Inject (Cell Biology Trading) software.

## Supporting Information

Figure S1 Knockdown of Ect2 results in binucleated cells. A. Ect2 siRNA reduce Ect2 levels in NIH3T3 total protein extract. Extracts from mock, Ect2 siRNA and non-specific siRNA (NS-siRNA) transfected cells analyzed by Western blotting. Only Ect2 siRNA transfected cells show reduction in protein level. B–D, Immunostaning with anti-α-tubulin and anti ECT2 antibodies show that while in the mock and the siRNA non-specific transfected cells ECT2 level was not affected, ECT2 siRNA transfected cells had much lower levels of ECT2. Commonly, these cells were also bi-nucleated as they fail to undergo cytokinesis.
(2.15 MB TIF)

Movie S1 Nocodazole leads to almost instantaneous microtubule de-polymerization. NIH3T3 cells stably expressing mCherry-α-tubulin subjected to nocodazole treatment. Microtubule disruption occurs within 4–8 minutes of nocodazole addition (108–116 min).
(1.29 MB AVI)

Movie S2 Non adherent DT40 cells contract throughout the cell cycle in a microtubule-dependent manner. A. DT40 cell undergo mitosis. B. Asynchronous culture treated with nocodazole contracts vigorously.
(3.49 MB AVI)

Movie S3 Detached adherent cells contract and fail to re-attach in a microtubules- dependent manner. NIH3T3 cells detached by trypsinization and allowed to re-adhere on a glass cover slip. Control cells (left panel) flatten within 30–60 minutes. Nocodazole-treated cells (right panels) contract and fail to re-adhere for at least 2 hours and usually flatten only after 4 hours.
(2.26 MB AVI)

Movie S4 Microtubules inhibit contractility in NIH3T3 mitotic cells. A, NIH3T3 cells stably expressing mCherry-α-tubulin undergo normal mitosis within an hour. B, Nocodazole was added one hour after the beginning of the movie. Seven hours later the cell detached from the plate, entered mitosis and immediately started to contract for more than 7 hours.
(8.07 MB AVI)

Movie S5 The mitotic spindle in cells arrested by nondegradable Cyclin B1 inhibits contractions in a spatial dependent manner. A, NIH3T3 cells stably expressing mCherry-α-tubulin were transfected with Cyclin B1-DM-GFP. Following mitotic entry and spindle formation (arrow) a cell portion, which was distal to the spindles (arrowhead) started to contract. When the spindle approached the distal part the contractions stopped (224–280 min). Phase (left panel), mCherry-α-tubulin (central panel), Cyclin B1-DM-GFP (right panel). B, NIH3T3 cells stably expressing mCherry-α-tubulin were transfected with Cyclin B1-DM-GFP, as described in A. The part of the movie presented here starts 20 hours after mitotic entry. After an additional 8.5 hours the cells were treated with nocodazole, which led to prompt spindle depolymerization and to vigorous contractions.
(5.97 MB AVI)

Movie S6 Inhibition of the RhoA-ROCK signaling pathway stops nocodazole induced contractions. NIH3T3 cells stably expressing mCherry-α-tubulin were transfected with dnRhoA-GFP and treated with nocodazole. A dnRhoA expressing cell (arrow) entered mitosis as indicated by the nuclear envelope breakdown (620 min), remained arrested for 5 hours and did not contract. Note the non transfected adjacent cell that entered mitosis earlier (568 min) and vigorously contracted.
(6.23 MB AVI)

Movie S7 Nocodazole induced contractions depend on proteasome activity. A. NIH3T3 cells stably expressing mCherry-α-tubulin were treated with both nocodazole and the proteasome inhibitor MG132. Nocodazole was added 4 hours before the beginning of the movie. MG132 was added at the beginning of the movie while the cell was already arrested and contracting. The cell continued to contract for an additional 7.5 hours and then ceased and remained as an arrested round mitotic cell. B. NIH3T3 cells where arrested for 15 hours with MG132 and nocodazole. They were subsequently washed and released into fresh medium without inhibitors. The cells seem to recover well and attach within about 2 hours.
(7.43 MB AVI)

Movie S8 Nocodazole induced contractions depend on ubiquitination. A31N-wt and A31N-ts20 cells were subjected to nocodazole at the permissive and restrictive temperatures. A, A31N-wt control cells undergo normal mitosis at 39.5°. B, An A31N-wt cell grown at 39.5° was treated with nocodazole and contracted upon mitotic entry. C, An A31N-ts20 cell grown at 34°, was treated with nocodazole. The cell contracted for 7 hours before it flattened. D, An A31N-ts20 cell was grown at 39.5° in the presence of nocodazole for 13 hours before it entered mitosis (788 min). During the next 14 hours the cell remained arrested, did not contracted and did not flatten.
(10.08 MB AVI)

Movie S9 Nocodazole induced contractions depend on neddylation. E36-wt or E36-ts41 cells were treated with nocodazole at the permissive and the restrictive temperatures. A, E36-wt control cells undergo normal mitosis at 39.5°C. B, An E36-wt cell grown at 39.5°C was treated with nocodazole and contracted upon entry into mitosis. C, An E36-ts41 cell grown at 34°C was treated with nocodazole. The cell arrested and contracted for 4 hours before it flattened. D, E6-ts41 cells undergo cell cycle arrest at 39.5°C. The movie started at 34°C and the cell (arrow, 44 minutes) underwent normal mitosis. Fifteen hours later the temperature was shifted to 39.5°C for an additional 11 hours. The cell was overall arrested for at least 26 hours. The cell cycle time of these cells at 34°C is about 16 hours. E, Temperature shift to 39.5°C of nocodazole-treated, mitotic E36-ts41 cells stopped their contractions. At the beginning of the movie the temperature was shifted to 39.5°C and nocodazole was added. Upon mitotic entry the cell (arrow, 52 minutes) contracted for 14.5 hours (944 minutes), and then stopped for an additional 4 hours. F, Detached control E36-wt (left panel) and E36-ts41 (right panel) were allowed to re-adhere on a glass cover slip. Nocodazole was added and the temperature was shifted to 39.5°C at the beginning of the movie. While control cells contracted and re-adhered within 4–8 hours, E36-ts41 cells contracted for 10 hours and then ceased. These cells failed to adhere the matrix.
(10.17 MB AVI)

Movie S10 Neddylation is upstream of the RhoA pathway. E36-ts41 cells were transiently transfected with constitutively active form of RhoA Q63L fused to GFP(caRhoA-GFP), grown at 39.5°C and treated with nocodazole. While the non-transfected control cell on the left panel did not contract (see also movie 9E) the transfected cell on the right panel vigorously contracted for over 14 hours. This indicates that the neddylation process required for contractility is upstream to the RhoA signaling pathway. Moreover, this suggests that prolong blocking of neddylation does not result in ATP depletion or in over activation Myosin phosphatase.
(5.42 MB AVI)
