# Supplementary figures and images for: Cellular Contractility Requires Ubiquitin Mediated Proteolysis
Source: PLoS One. 2009 Jul 14;4(7):e6155. doi: 10.1371/journal.pone.0006155 (PMC2705188; doi:10.1371/journal.pone.0006155)

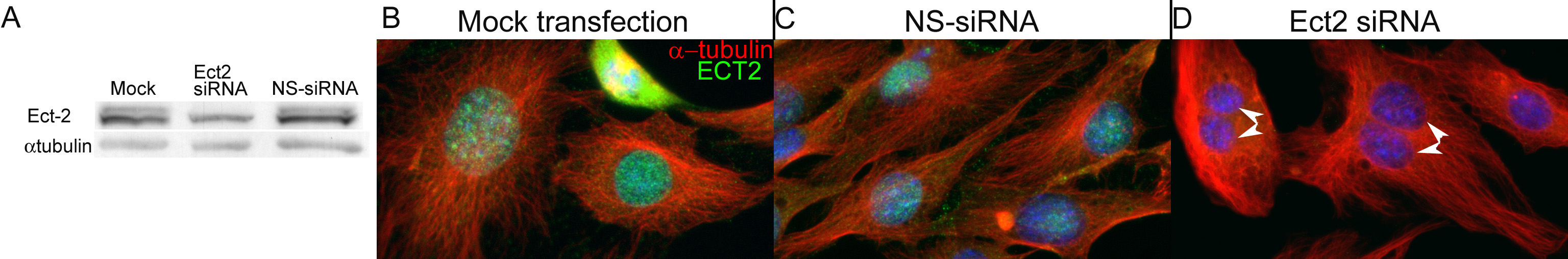

Supplement: Figure S1 — Knockdown of Ect2 results in binucleated cells. A. Ect2 siRNA reduce Ect2 levels in NIH3T3 total protein extract. Extracts from mock, Ect2 siRNA and non-specific siRNA (NS-siRNA) transfected cells analyzed by Western blotting. Only Ect2 siRNA transfected cells show reduction in protein level. B–D, Immunostaning with anti-α-tubulin and anti ECT2 antibodies show that while in the mock and the siRNA non-specific transfected cells ECT2 level was not affected, ECT2 siRNA transfected cells had much lower levels of ECT2. Commonly, these cells were also bi-nucleated as they fail to undergo cytokinesis. (2.15 MB TIF) [file pone.0006155.s001.tif]
